# Editorial: Deep learning for high-dimensional sense, non-linear signal processing and intelligent diagnosis

**DOI:** 10.3389/fpsyt.2024.1538534

**Published:** 2025-01-10

**Authors:** Hengjin Ke, Cang Cai, Jia Wu, Dan Chen

**Affiliations:** ^1^ School of Computer, Hubei Polytechnic University, Huangshi, China; ^2^ Faculty of Artiffcial Intelligence Education, Central China Normal University, Wuhan, China; ^3^ School of Computing, Macquarie University, Sydney, NSW, Australia; ^4^ School of Computer Science, Wuhan University, Wuhan, China

**Keywords:** deep learning, non-linear, intelligent diagnosis, interpretation, medical image

## 1 Introduction

In recent years, deep learning techniques (1) have transformed the landscape of high-dimensional signal processing and intelligent diagnostics. Given the rising demand for efficient and accurate systems in fields such as medical imaging, neuroinformatic (2), and various high-stakes diagnostic applications, this Research Topic delves into advances in non-linear processing methods that address the complexity of real-world signals. The unique contributions in this Research Topic underscore how cutting-edge approaches in neural networks (3), particularly those tailored for high-dimensional and nonlinear data such as EEG, MRI and Perfusion-CT (4), are reshaping diagnostic technologies and interpretability.

## 2 Objectives of the Research Topic

The aim of this Research Topic is to gather significant studies that explore the intersection of deep learning, non-linear signal processing, and intelligent diagnostic applications. Each contribution addresses a unique facet of these complex domains, shedding light on innovations and revealing insights into the potential and limitations of deep learning methods in high-dimensional contexts.

## 3 Summaries of key contributions

### 3.1 Deep learning for medicine interpretation

#### 3.1.1 Enhancing interpretability in autism research

Chen et al. focus on improving the understanding of neural network models in autism classification. By integrating deep symbolic regression with the Deep Factor Learning model on a Hilbert Basis tensor [HB-DFL (5)], they extract non - linear factors from fMRI data. This approach allows for a dynamic interpretation of factor matrices and the construction of brain networks for in - depth analysis. The study’s findings contribute to a better understanding of the pathophysiology of autism, providing a foundation for more informed treatment and intervention strategies.

#### 3.1.2 Insights into MDD classification

Zhou et al.’s work on using a TanhReLU - based Convolutional Neural Network (CNN) for Major Depression Disorder (MDD) classification not only achieves high accuracy but also offers insights into the model’s behavior. The stable learning curves and high-performance metrics provide evidence of the model’s generalizability, which is crucial for its potential application in clinical settings. This research contributes to the field by demonstrating how an innovative activation function can enhance the interpretability and effectiveness of deep learning models in mental health diagnostics.

### 3.2 Application to medical imaging diagnostics

#### 3.2.1 Innovative approaches for autism diagnosis

Shi et al. introduce an autoencoder - based method for generating brain MRI images of patients with Autism Spectrum Disorder (ASD) and Healthy individuals. The detailed architecture and training process of the autoencoder model, along with the subsequent classification using a convolutional neural network, offer a novel framework for ASD diagnosis. This approach not only addresses the challenges of limited sample sizes and high data heterogeneity but also provides a new perspective on the automated diagnosis of ASD using medical imaging data.

#### 3.2.2 Advancing depression treatment prediction

Lu et al.’s Graph Frequency Attention Convolutional Neural Network (GFACNN) for predicting depression treatment response from EEG signals represents a significant advancement in medical imaging diagnostics By transforming EEG signals into graphs and incorporating a frequency attention module, the model can effectively capture the complex relationships within the data. The experimental results on a real - world dataset demonstrate the model’s high accuracy and discriminative power, highlighting its potential to assist clinicians in making more informed treatment decisions.

#### 3.2.3 Innovative energy optimization models for predicting cardiovascular disease

A novel deep learning framework, the CGC-Net model, integrating Convolutional Neural Networks (CNN), Gated Recurrent Units (GRU), and a Contextual Self-Attention (CSA) mechanism, is proposed for predicting cardiovascular disease by analyzing patient behavior patterns. The model demonstrates superior accuracy and computational efficiency. Comprehensive experiments validate its advantages, providing a practical solution for improving building energy efficiency and reducing costs while supporting sustainable development in the construction industry (Wang et al.).

### 3.3 Advances in non - linear signal processing

#### 3.3.1 Autoencoder for MRI data analysis

Shi et al.’s use of an autoencoder in handling MRI data showcases its effectiveness in learning data representations, reducing noise, and enhancing the clarity of images. The ability of the autoencoder to handle high - dimensional neuroimaging data and capture essential features is a significant contribution to non-linear signal processing in the context of medical imaging. This work provides a valuable tool for analyzing complex MRI data and extracting meaningful information for autism research.

#### 3.3.2 Non-linear interpretability in deep learning

Recent advancements in deep learning have leveraged increased network complexity and non-linear transformations to achieve unprecedented success in diverse applications, including medical imaging and autism diagnosis. These non-linear methodologies have enabled deep neural networks to capture intrinsic patterns and dynamics in fMRI data, providing insights into the underlying neurobiological mechanisms of conditions like autism. Despite progress, challenges remain in enhancing model interpretability to ensure reliability and precision, particularly in medical contexts (Chen et al.). Hybrid approaches, such as combining deep learning with symbolic regression, and advanced tensor factorization techniques, like the HB-DFL, have demonstrated promise in extracting non-linear factors and elucidating dynamic model behavior, paving the way for accurate, interpretable, and dynamic analyses of neuroimaging data.

## 4 Conclusion and future directions

This Research Topic highlight how each article contributes to advancing deep learning applications in signal processing and diagnostics. Taken together, the findings suggest promising pathways for future exploration. Particularly, there is scope for exploring new approaches including temporal-spatial feature learning (6) with real-time diagnostic systems, multi-modal data fusion, and healthcare applications (7). We anticipate that this Research Topic will inspire ongoing innovation and spur collaboration within the research community.
